# Comparison of Two Xenograft Materials Used in Sinus Lift Procedures: Material Characterization and In Vivo Behavior

**DOI:** 10.3390/ma10060623

**Published:** 2017-06-07

**Authors:** María Piedad Ramírez Fernández, Patricia Mazón, Sergio A. Gehrke, Jose Luis Calvo-Guirado, Piedad N. De Aza

**Affiliations:** 1 Cátedra Internacional de Investigación en Odontología, Universidad Católica San Antonio de Murcia, Avda. Jerónimos, 135, 30107 Guadalupe, Murcia, Spain; jlcalvo@ucam.edu; 2 Departamento de Materiales, Óptica y Tecnologia Electrónica, Universidad Miguel Hernández, Avda. Universidad s/n, 03202-Elche, Alicante, Spain; pmazon@umh.es; 3 Biotecnos Research Center, Rua Dr. Bonazo n° 57, 97015-001-Santa Maria (RS), Brazil; sergio.gehrke@hotmail.com; 4 Instituto de Bioingenieria, Universidad Miguel Hernandez, Avda. Ferrocarril s/n. 03202-Elche, Alicante, Spain; piedad@umh.es

**Keywords:** hydroxyapatite, xenografts, physic-chemical-characterization, tissue reaction

## Abstract

Detailed information about graft material characteristic is crucial to evaluate their clinical outcomes. The present study evaluates the physico-chemical characteristics of two xenografts manufactured on an industrial scale deproteinized at different temperatures (non-sintered and sintered) in accordance with a protocol previously used in sinus lift procedures. It compares how the physico-chemical properties influence the material’s performance in vivo by a histomorphometric study in retrieved bone biopsies following maxillary sinus augmentation in 10 clinical cases. An X-ray diffraction analysis revealed the typical structure of hydroxyapatite (HA) for both materials. Both xenografts were porous and exhibited intraparticle pores. Strong differences were observed in terms of porosity, crystallinity, and calcium/phosphate. Histomorphometric measurements on the bone biopsies showed statistically significant differences. The physic-chemical assessment of both xenografts, made in accordance with the protocol developed on an industrial scale, confirmed that these products present excellent biocompatibilitity, with similar characteristics to natural bone. The sintered HA xenografts exhibited greater osteoconductivity, but were not completely resorbable (30.80 ± 0.88% residual material). The non-sintered HA xenografts induced about 25.92 ± 1.61% of new bone and a high level of degradation after six months of implantation. Differences in the physico-chemical characteristics found between the two HA xenografts determined a different behavior for this material.

## 1. Introduction

The use of dental implants for the rehabilitation of missing teeth has increased treatment options for patients. Loss of teeth in the posterior maxillary area can lead to adverse consequences. It is not uncommon to observe severe maxillary sinus pneumatization, which reduces the implant prosthetic alternatives to replace missing teeth. In this anatomical situation, it can be very difficult to obtain effective primary stability. Maxillary sinus augmentation has been shown to be a predictable method to increase posterior maxillary bone height, and allows placing dental implants when the residual alveolar ridge is deficient in bone volume [1].

Nowadays, there are several types of graft materials used in sinus lift procedures, each with its advantages and disadvantages [2]. The ideal bone grafting material should have both osteoinductive and osteoconductive properties. Osteoinduction is defined as primitive, undifferentiated, and pluripotent cells stimulated by inductive means to induce bone-forming cells and osteogenesis processes. Osteoconduction is defined as bone growth on a surface, which is called the osteoconductive surface, and allows the osteogenesis process itself down into pores [3].

Natural bone is a complex inorganic-organic nanocomposite material in which hydroxyapatite [Ca_10_(PO_4_)_6_(OH)_2_] nanocrystallites and collagen fibrils are well organized in hierarchical architecture overall length scales, including nanoscales. Hydroxyapatite (HA) is the major inorganic component of natural bone and has been applied widely in the medical field as a bone repair material given its excellent bioactive and biocompatibility properties. HAs are known for being biocompatible and bioactive (ability to form a direct chemical bond with surrounding tissues; ossteoconductive; and non-toxic, non-inflammatory, and non-inmunogenic properties) [4].

Technological evolution, along with a better understanding of bone-healing biology, has led to the development of several bone grafts [5]. Biomaterials that mimic the structure and composition of bone tissue on the nanoscale are important for the development of bone tissue engineering applications. Synthetic hydroxyapatites are the most widely used [6], but do not completely match the chemical composition of human bone [7]. After the increasing application of synthetic HAs in the last decade, a shift back to natural HAs has taken place [8].

Fortunately, bones from other species possess a similar tissue structure to that of human bones. These bones also exhibit osteoinduction and osteoconduction activities, which potentially satisfy the requirements of ideal bone graft substitutes [9]. Information from all reviews does, however, substantiate the finding that implant survival with bone replacement grafts, especially the most rigorously evaluated group (xenografts), equals or betters those achieved with autogenous bone [10]. These data, coupled with lower patient morbidity achieved by eliminating the need for a secondary surgical (donor) site, appears to position bone replacement grafts as the graft material of choice today. The histologic results with xenografts present a pattern that has been called “bone bridging”. Residual xenograft particles are surrounded partly by new vital bone, and are joined to nearby particles through this mechanism [11].

The availability of bovine bone is practically unlimited, and possesses a great physic-chemical and structural similarity to human bone [12]. For many years now, bovine cortico-spongy bone has been the first choice for buccal and maxillofacial surgery. There are numerous reports in the literature which have observed successful regenerative procedures in patients treated with maxillary sinus augmentation using bovine bone [13,14,15,16,17].

Other xenograft materials of other origins have been introduced into the clinical and research fields of dentistry under the banner of preventing the bovine-specific disease transmission [18]. Deproteinized porcine bone mineral is a substitute for deproteinized bovine bone mineral, and several researchers have already introduced it into clinical dental procedures based on the structural/physiological similarities of bone tissue between human and swine [19]. These xenografts have led to a great deal of research thanks to their potential as an adequate bone substitute for maxillary sinus augmentation [20,21,22].

Eliminating antigens that potentially cause an immune response is a prerequisite for xenogenic bone grafting. It has been shown that deproteinized bones not only lose their immune reactivity, but also retain their osteoinduction and osteoconduction activities [23].

The physico-chemical properties and mechanical strength of deproteinized tissue-engineered bone meet the demands of ideal scaffold materials. In the bioceramics field the need to correlate the chemical and physical composition of HA with cellular interactions still remains [24].

It has been assumed that the difference between filling materials used in sinus elevation procedures may modulate the quality and quantity of newly formed bone. The selection of biomaterials constitutes a key point for successful tissue regeneration practice. The mechanisms behind tissue response to HAs have not yet been comprehensively established [25].

All substitute material aspects must be studied. Some studies have demonstrated that osteointegration and degradation processes are influenced by the material’s physico-chemical properties [26], including granule size [12], morphology [27], crystallinity [28], porosity [29,30,31], surface roughness [32] and the calcium/phosphate ratio in the composition [33,34]. Part of a biological response produced by a biomaterial is conditioned by its physico-chemical properties. The work reported herein indicates the vast importance of fully characterizing the materials used. The many alternatives available, compared to the few comparative studies conducted, leave the choice of grafting material to the surgeon´s preference, and are not always scientifically-based [34].

Based on these data, the present study developed a protocol for the characterization of xenografts. The objective of this study was to characterize the physico-chemical properties of two xenografts deproteinized at different temperatures, and to know how the physico-chemical properties influence the material’s performance in vivo.

## 2. Results

### 2.1. Graft Implants Characterization

#### 2.1.1. Scanning Electron Microscopy-Energy Dispersive X-ray Spectroscopy (SEM-EDX) Analysis

**Figure 1:**
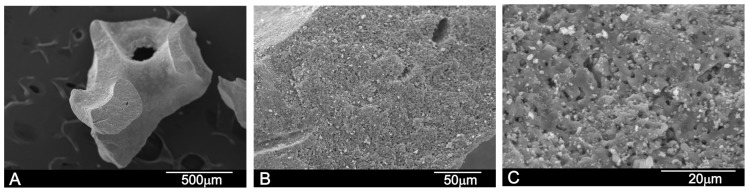
Scanning electron micrographs of the Bovine HAs scaffolds deproteinized at high temperature prior to the insertion for maxillary sinus floor elevation (BBM): (**A**) low magnification; and (**B**) high magnification showing macro- and microporosity; and (**C**) details of the microporosity on the scaffold surface together with apatite crystals (white).

**Figure 2:**
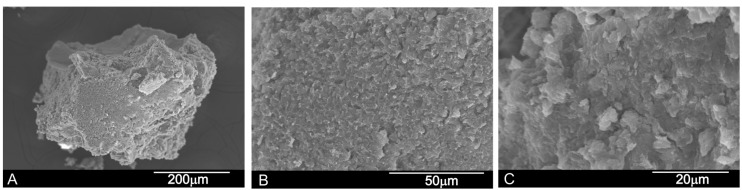
Scanning electron micrographs of Porcine HAs scaffolds deproteinized at low temperature (PBM): (**A**) low magnification image of the HA grafts; (**B**) high magnification image; and (**C**) details of the scaffold’s surface roughness.

The SEM micrographs provided information about the morphology of the obtained bovine HAs scaffold (Figure 1) and the porcine HAs scaffolds (Figure 2). The bovine HAs scaffold (called BBM from this point onward) consisted of a highly porous network with an average pore size of 0.5 mm (Figure 1A). Micropores from 1 μm to 5 μm were also visualized. When granules were evaluated at larger magnifications, it was possible to observe their porous surface roughness with apatite crystals (white particles) (Figure 1B,C).

The porcine HAs scaffold (called PBM from this point onward) consisted of small grains of 500 μm on average (Figure 2A). At high magnification, the PBM shows surface roughness (Figure 2B). Due to the presence of collagen, the material shows no microporosity on its surface (Figure 2B) at high magnification, and the surface is not as clear as in the BBM xenograft.

EDX was used to determine the elemental composition in an area reached from graft particles before implantation. The Ca/P ratios for both the HA xenografts were 2.31 ± 0.09 for the BBM material and 2.22 ± 0.08 for the PBM material. The Kolmogorov–Smirnov test rejected Normality for the PBM group. A statistically significant difference was found between BBM and PBM (Mann–Whitney test, *p* < 0.0051).

#### 2.1.2. X-ray Diffraction Analysis (XRD)

**Figure 3:**
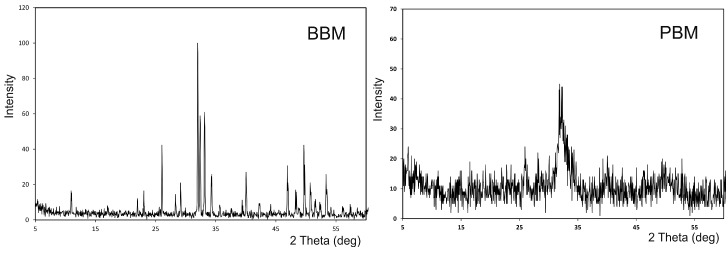
X-ray diffraction pattern of the BBM and PBM materials.

Figure 3 shows the X-ray diffraction patterns of the BBM and the PBM materials. The XRD of the grafts can be associated with the chemical composition of samples. 

As expected, the XRD pattern from the mineral samples corresponded to HA, with coincident peak positions and relative intensities. However, these materials presented diverse degrees of crystallinity, as indicated in the different peak widths. That occurred with PBM, as the diffractogram exhibited broad peaks with a low signal-to-noise ratio, which corresponded to a low-crystallinity material. The sharp and well-resolved peaks found in the XRD spectrum of BBM indicated a highly crystalline HA. The HA corresponded to JCPDS card no. 09-0432 and presented a hexagonal system with a main diffraction plan [211]. No other secondary phases were detected.

#### 2.1.3. Mercury Intrusion Porosimetry (MIP) Analysis

**Figure 4:**
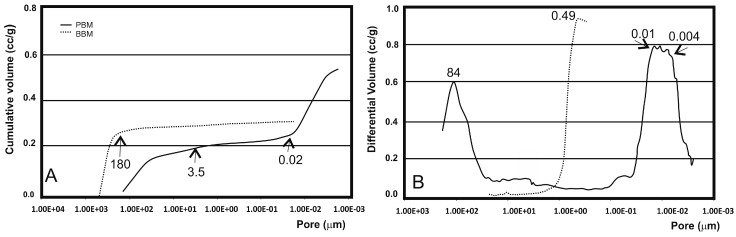
Mercury intrusion curves of the PBM and BBM ceramics measured by mercury porosimetry: (**A**) cumulative intruded volume versus pore diameter; and (**B**) differential-intruded volume versus pore diameter.

When analyzing a granular material by mercury porosimetry, two kinds of spaces can be detected: those that correspond to the empty spaces between particles (commonly designated by “interstices” or “interparticle” spaces) and those that correspond to the spaces of the particles themselves (known as “pores” or “intraparticle” spaces). The results obtained for the granules of PBM (Figure 4) showed that with increasing pressure, mercury penetrated to the increasingly smaller pores. The cumulative curve (Figure 4A) denotes a small intrusion in the pores between 300 μm and 3.5 μm, followed by a plateau between 3.5 μm and 0.02 μm where no intrusion is detected, and then significant mercury penetration into the pores below this value. The initial rise of the curve corresponds mostly to the filling of the spaces between particles (and may also include some of the largest pores of cancellous bone), whereas the later stage of rise is related to the pores of individual particles. The range of the intraparticle pores is more obvious in (Figure 4B) in which one intense peak, whose mode falls within the 0.01–0.004 μm range, is clearly visible. The peak on the left (84 μm) corresponds to the intrusion of mercury in the interparticle spaces. The size of these spaces, related to the way that particles are packed, depends on both particle size and shape, as well as one particle size distribution. However, the distinction made between inter- and intraparticle spaces is not always so apparent. This interpretation aimed to elucidate the kind of information that can be extracted from pore size distribution curves and to highlight the importance of always specifying the size range of measured pores. It should be stressed that the mercury intrusion technique is especially suited for analyzing intraparticle pores, but is not adequate for measuring large spaces (300 μm). 

The results obtained for the granules of BBM denoted mercury intrusion in the pores between 300 μm and 180 μm, followed by a plateau between 180 μm and 0.02 μm where no intrusion was detected. The range of the intra- and inter-particle pores is not obvious in (Figure 4B), in which only one intense peak, whose mode is about 0.49 μm, is clearly visible in the interparticle spaces, but the proportion of interparticles in BBM is clearly lower than in PBM. 

Table 1 summarizes these results in terms of the total intruded volume, mode of intraparticle pores, total porosity and intraparticle porosity (taken as the percentage of the particles’ internal pores <1 μm in relation to total porosity). An analysis of Table 1 suggested that PBM had the greatest porosity (59.90%). However, about 40% (38.11%) of this porosity corresponded to submicron pore entrances. A similar porosity value was determined for the BBM samples. This also exhibited a much smaller proportion of submicron pores, with only 3.66%.

### 2.2. Clinical and Radiographic Results

Dropouts were not observed during evaluation period and all returned for implant placement. Both groups presented similar baseline characteristics in terms of alveolar bone height values. All patients were operated on successfully. 

**Figure 5:**
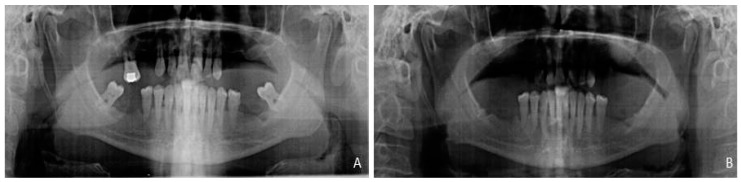
(**A**) Preoperative panoramic image before the sinus lift; and (**B**) postoperative panoramic image 6 months after the sinus lift.

After a six-month follow-up period of these 10 partially edentulous patients treated with xenograft materials for sinus floor augmentation, the success rate was 100%. No sinus membrane perforation or other clinical complications, such as sinusitis or pain, resulted from surgery. The increased volumes produced by the xenograft procedures were stable by the end of the healing period. The radiographic findings showed that both treatment modalities resulted in bone gain at six months in both groups. The increase in alveolar bone height scores was evident between pre-augmentation and 6 months after in both groups. All 10 augmented sinuses provided 12 mm or more available bone for implant placement, as seen in (Figure 5).

**Figure 6:**
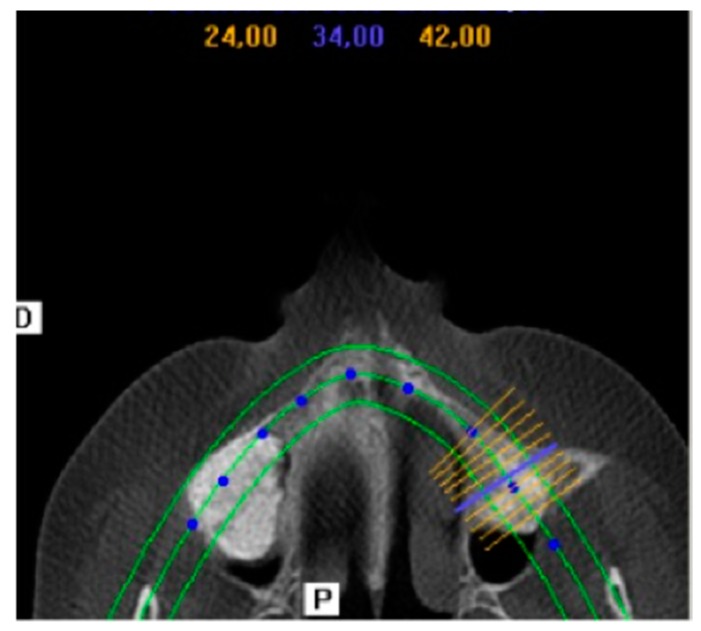
i-CAT (Imagen-computerized axial tomography) Vision postoperative image. It shows increased volumes produced by the xenograft procedures after six months of maxillary sinus elevation with a porcine hydroxyapatite (cuts 24–42).

Bone density in the grafted area with both biomaterials was evaluated radiologically as a routine diagnostic approach (Figure 6). At the moment of implant insertion, after a healing period of six months, the augmentation sites treated with the PBM show less dense new bone formation achieved along the inner surface of replaced bony widow than the area on the bone graft whilst the BBM shows that more dense new bone formation was achieved in the area on the bone graft respect the original augmentation density. Bone area was different in the groups after implantation and increased with time. Bone initially formed at the sinus wall and proliferated into the center of the augmented sinus cavity. Three implants were not osseointegrated at the end of the study, leaving 57 implants for control (95% rate of success).

### 2.3. Histomorphometric Results

#### SEM-BSE Analysis

**Figure 7:**
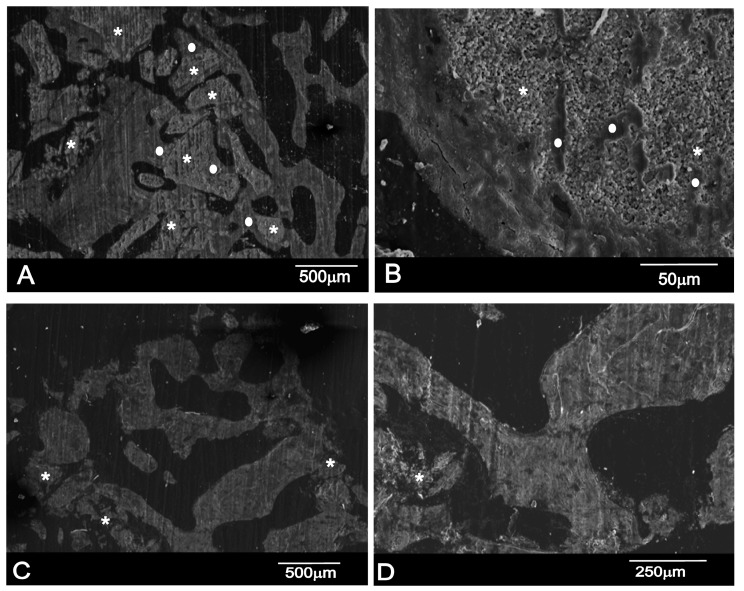
SEM-BSE of a resin-embedded bone section that contains bone and residual biomaterials: (**A**,**B**) the BBM material; and (**C**,**D**) the PBM material (* material particles as a result of the degradation process, and • new bone). Section of the bone retrieved from a patient’s maxillary sinus six months after sinus elevation.

A section of the material–bone interfaces six months after sinus elevation is shown in Figure 7. The Scanning Electron Microscopy with Back-Scattered (SEM-BSE) evaluation confirmed that the residual graft particles were surrounded by newly formed bone, which presented mature bone characteristics with well-organized lamellae (Figure 7). The BBM material shows progressive structure dissolution, and the result of these processed free graft particles were found in many areas. Asterisks (*) and bullets (•), respectively, denote the graft particles and ingrown bone region in Figure 7A,B.

In many fields, it was observed that the majority of the residual graft particles were connected by bridges to new bone. The bone-to-biomaterial interface was characterized by numbers of projections of newly formed bone into the graft particles. In most cases, the particle perimeter appeared to be lined by bone that adhered tightly to the biomaterial surface; although some bone was detached from the particle surface, this was only in a few very small areas. In backscattered electron images, particles of hydroxyapatite crystals were seen to be a white-gray color due to low organic content and a relatively high Ca/P ratio, whereas newly formed bone had a darker gray color because of the presence of collagen, marrow, and fat. 

The PBM material degrades faster and, at the same time, new bone is reabsorbed, so we can see only material particles (*) surrounded by non-mineralized connective tissue (Figure 7B,C).

**Figure 8:**
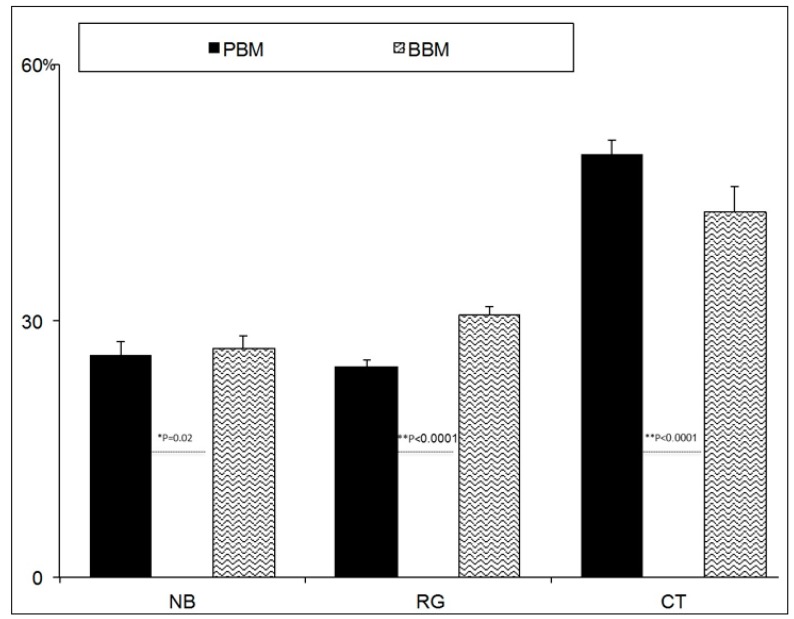
Histomorphometric measurements on bone biopsies. Histomorphometry shows the percentage of newly formed bone, marrow spaces and the residual grafted material. The figure provides the mean and SD values for all the parameters in each group. Statistically significant differences were seen when comparing BBM and PBM in relation to NB (new bone) (Mann–Whitney test, * *p* = 0.02), RG (residual graft material) (Mann–Whitney test, ** *p* < 0.0001), and CT (connective tissue) (Mann–Whitney test, ** *p* < 0.0001).

In histomorphometric terms, the BBM group showed that newly formed bone had become closely attached to both HAs. The histomorphometric measurements on the bone biopsies showed that, for PBM, the newly formed bone represented 25.92 ± 1.61%, and 24.64 ± 0.86% for the residual graft material and 49.42 ± 1.62% for connective tissue, while 26.83 ± 1.42% represented the BBM newly formed bone, 30.80 ± 0.88% for the residual graft material, and 42.79 ± 2.88% for the non-mineralized connective tissue. Statistically significant differences were seen when comparing BBM and PBM in relation to NB (Mann–Whitney test, *p* = 0.02), RG (Mann–Whitney test, *p* < 0.0001), and CT (Mann–Whitney test, *p* < 0.0001). The mean and standard deviation (SD) values for all the parameters in each group are found in (Figure 8).

## 3. Discussion

In the present study, two different xenogenic bone substitute materials were characterized and evaluated in vivo in sinus floor elevation procedures. This study compared BBM processing at high temperature (1200 °C) and PBM processing at low temperature (130 °C) with different physico-chemical properties as bone substitutes for healing in sinus lift procedures. In this paper, the effect of sintering temperature on the porosity, crystallinity, composition and phase purity of porous HA ceramics made of natural bone was studied and reported. The characterization of HAs ensured an overall understanding about the role played by the biological behavior of these bone substitutes. Moreover, further importance is attached to this research because of the scarce scientific documentation in the literature comparing BBM and PBM in sinus lift procedures.

This study demonstrated that sinus augmentation with both BBM and PBM produces an increased vertical bone dimension compared to the baseline values, which accommodates dental implant placement. 

Natural HA grafts may be a suitable alternative for using autogenous grafts. However, using these materials is sometimes avoided through lack of information, or due to contradictions between manufacturers’ specifications and clinical results [9]. The HA derived from either natural sources or synthetic sources can form a strong chemical bond with the host bone tissue. This allows it to be recognized as a good bone substitute material. Xenografts have different properties depending on their origin, constitution and processing. Those that usually come from cows and pigs could be osteoinductive and osteoconductive, low-cost and offer good availability, but have the disadvantages of immune response and the risk of transmitting animal diseases. Significant differences in the use of biomaterials of animal or synthetic origin have yet to be reported. Some evidence suggests that synthetic materials have a lower risk of disease transmission [35]. Deproteinization is an indispensable process to eliminate antigenicity in xenograft bones. Valid strategies to eliminate the antigenicity of xenograft bones are of vital importance in the development of xenogenic bone graft substitutes [36]. It has been shown that deproteinized bones not only lose their immune reactivity, but also retain their osteoinduction and osteoconduction activities [23]. Sintering temperature is seen as an important factor that can alter the characteristics of HA [37]. However, the effect of sintering temperature on the physico-chemical properties of natural HA, especially HA from bovine bone, is still not fully understood and research into this area is still underway. The most important parameters that can affect the properties of HA are temperature and heat treatment duration [38]. Both used materials differ significantly in processing temperature terms. Our results showed that the examined HA had variable physico-chemical properties according to the study of Muralithran and Ramesh’s [39]. This discrepancy may affect the materials’ performance, and indeed significant differences were found in terms of new bone formation, residual graft material and non-mineralized connective tissue after six months of healing.

The extensive characterization of the graft materials BBM and PBM made herein revealed that these two commercial bone grafts, despite being used in the clinical practice for the same purposes, possess markedly different properties, which are both chemical (e.g., composition, crystallinity, Ca/P ratio) and physical (e.g., particle size and shape, and pore size distribution). It is thus not surprising to find that they induce different responses after sinus lift elevation. The differences found in the explored characteristics seem to justify this distinct in vivo performance. Some studies have demonstrated that osteointegration and degradation processes are influenced by the material’s physical and chemical properties [12,27,28,29,30,31,32,33,34]. Despite different responses being somehow anticipated since these materials, as the results show, have quite distinct properties, the following discussion attempts to interpret the in vivo response of these two biomaterials in terms of their physico-chemical characteristics.

The histologic examination of 60 biopsies taken from the 10 patients in this study indicated statistically significant differences in the histomorphometric parameters between the BBM and PBM graft materials. In the present study, a histomorphometric analysis of the biopsies done after six months showed a percentage of newly formed bone, which represented 25.92 ± 1.61% and 24.64 ± 0.86% for the residual graft material and 49.42 ± 1.62% for the connective tissue for PBM, while, for BBM, newly formed bone represented 26.83 ± 1.42%, with 30.80 ± 0.88% for residual graft material and 42.79 ± 2.88% for non-mineralized connective tissue. New bone formation methods for bone deproteinization have to allow the heterologous deproteinized bone to have good biological safety and to meet all the scaffold material demands of tissue engineering. Moreover, the quantity and quality of newly formed bones must improve. The trabecular porous architecture of deproteinized bones acts as a support structure for blood vessels and bone cell expansion, which are extremely important for ossification [24]. PBM obtained the greater porosity 59.90%, but 38.11% of this porosity corresponded to submicron pore entrances. BBM porosity was 49.13%, but exhibited a much smaller proportion of submicron pores, only 3.66%. If pore size was too small, vascular supply could be compromised and osteoconduction decreased. This study confirmed the findings of earlier studies, which have reported the relation between pore and the quantity of neoformed osseous tissue. They concluded that pore size and granulometry should not be over-reduced as both pore diameter and interporotic connections have a significant effect on the type and quantity of newly formed bone tissue [40,41,42,43].

Contrarily to a similar radiological and histomorphometric design study at 8 months after a sinus lift procedure of two xenografts of bovine bone origin deproteinized at different temperatures, Deproteinized bovine bone (DBB-1) deproteinization occurred at low heat (300 °C) and (DBB-2) at a very high temperature (1200 °C), and no significant differences were found in new bone formation and residual graft material. In the histomorphometric analysis, new bone formation and residual graft materials were 29.13% and 24.63%, and 14.77%, and 13.01% in the DBB-2 + Collagen membrane (CM) and DBB-1 + CM groups, respectively. Although the healing period was two months longer in the study by Panagiotou et al. [44], we agree with their study in that the particles related to both graft materials were still observed in specimens, which demonstrates the slow resorption rate of the BBM materials. Since DBB-1 has a less crystalline structure compared to DBB-2, and might be more prone to degradation, residual DBB-1 particles might resorb more quickly. In our study into the characterization of both Has, the XRD patterns from the mineral samples corresponded to HA, with coincident peak positions and relative intensities. However, these materials presented diverse degrees of crystallinity, as indicated by the different peak widths. For PBM, the diffractogram exhibits broad peaks with a low signal-to-noise ratio, which corresponds to a low-crystallinity material. The sharp well-resolved peaks found in the XRD spectrum of BBM indicate a highly crystalline HA. Thus, PBM sinterized ar low temperature acquires a less crystalline structure compared to the BBM sinterized at high temperature, and might also be more prone to degradation. Tissues respond differently to biomaterials of different crystallinities. Major differences in the adhesive response of epithelial cells and osteoblast precursor cells to different crystallographic structures have been reported [27].

Regarding the sintering characteristics of HA, the resulting microstructure and properties were influenced by thermal history during the fabrication process. In the present study, the results showed that sintering temperature was a critical factor which influenced the phase stability, densification behavior, crystallinity and porosity of bovine HA ceramics. According to the results of research on the effect of sintering temperature, the density and hardness of HA increases with a rising sintering temperature, according to the results obtained [38].

A similar study that compared the sintered and non-sintered bone substitute materials in the sinuses of eight patients has indicated no significant differences in new bone formation and residual graft material terms. The examined xenogenic bone substitute materials were both gained with the same bovine origin. They differed mainly in the deproteinization way. The histological analysis revealed comparable results for the sintered (SBM) and non-sintered xenogeneic bone substitute material (NSBM). At the sites treated by sintered materials 29.71 ± 13.7% of the augmented site consisted of new bone, whereas the percentage of new bone was 30.57 ± 16.1% in the non-sintered group, together with the percentage of bone substitute materials in the augmented area (40.68 ± 16.3% for the sintered group, and 43 ± 19.1% for the non-sintered group) [45]. Even the histomorphometric results revealed a lower percentage of residual graft particles for the sintered group compared with our results.

According to other studies, which have also compared two xenograft materials prepared by a deproteinizing technique at low temperature (Bio-Oss) or high temperature (Cerabone) in the sinus cavity, compared to our study they observed a significantly greater volumetric loss of the initial graft size for the non-sintered material. Bio-Oss has a significantly larger surface area and a smaller crystallite size compared to Cerabone. This might have a crucial influence on the resorption rate [46]. In the BBM process, an increasing sintering temperature caused increased sample crystallinity and induced HA densification, which resulted in grain growth with the formation of dense grain boundary phases. These differences were associated with significantly higher resorption rates for the initial graft volume observed for the PBM material. Depending on the degrees of crystallinity, albumin solution or cell suspension has been suggested to selectively adsorb on HA surfaces as such, and this study demonstrated the importance of determining the effects of the physico-chemical characteristics of HA surfaces on cell activity [28]. Dissolution of HA is dependent on its crystallinity, with dissolution increasing with lowering degrees of crystallinity. Albumin adsorption and cell attachment are seen to selectively adsorb on the HA surface, and the degree of adsorption is dependent on HA crystallinity [47].

Properties like solubility and surface reactivity are highly dependent on calcium phosphate composition and surface texture. Such characteristics strongly affect the nature of the biologically equivalent (carbonated) apatite formed when calcium phosphates come into contact with bone tissue [48]. In our study, a statistically significant difference was found between PBM 2.22 ± 0.08 and BBM 2.31 ± 0.09 in the calcium/phosphate composition ratio. The slow dissolution capacity of the BBM graft was attributed to the small quantity of tricalcium phosphate produced during heat treatment. The annealing temperature affects the type and amount of other calcium phosphate phases and/or other Ca compounds, which are present with the HA phase [49].

Deproteinized bones at low temperature have shown lower protein content, and a higher collagen content has been preserved [24]. The Tecnoss patented manufacturing process, used to produce PBM, achieves biocompatibility by avoiding temperatures above 120 °C, which would cause the ceramization of granules bypreserving part of the collagen matrix of the original animal bone. The result was a unique biomaterial that consisted of mineral components and an organic matrix with a very similar level of porosity to autogenous bone that quickly resorbs. The role of the collagen composition of the PBM used herein is useful for its intrinsic agglutination characteristic, which help in structuring the composite, influence the morphology and size of HA crystals and, at the same time, contribute in the process of osteoclast adhesion to the biomaterial surface [4,50]. 

It has been hypothesized that the hydrolytic enzymes (collagenase) released from activated PMN cells are involved in the rapid degradation of the graft’s porcine collagen portion. However, no studies have reported the outcome of this material after maxillary sinus floor elevation [51].

The aforementioned properties of the graft material have led researchers to choose bovine bone material as the gold standard of xenografts because it has been demonstrated to be a biologically inert osteoconductive material for sinus augmentation procedures [52].

Biocompatibility, osteoconduction and low rate resorption after surgery are favorable properties of xenografts, but long-term studies must be carried out to understand xenografts’ pattern of biodegradation and its influence on bone gain [50]. 

## 4. Materials and Methods

### 4.1. Grafts

Two different types of commercial bone grafts used in dentistry were characterized and evaluated in vivo in relation to the bone tissue response:

1. (PBM, OsteoBiol^®^ mp3 deproteinized porcine bone substitute material) was made up of granulated small bone particles, 600–1000 μm in size, and is a ceramic that derives from cancellous-cortical porcine bone. The material is obtained at a low processing temperature (130 °C). According to its commercial specifications, this material is claimed to preserve the structure and composition of natural bone components.
2. (BBM, Endobon^®^ deproteinized bovine bone substitute material) was made up of granulated small bone particles, 500–1000 μm in size, and is a ceramic that derives from cancellous bovine bone, which is fully deproteinated by a high temperature manufacturing process for safety from bacteria, viruses and prions. This process consists of two steps: firstly, pyrolysis at a temperature above 900 °C to eliminate the organic element; secondly, a ceramization process at temperatures above 1200 °C to creates a crystalline structure.

### 4.2. Graft Characterization

These biomaterials were characterized in morphology, composition, crystallinity, particle size distribution, porosity and pore size terms. 

#### 4.2.1. SEM-EDX

An analysis using SEM-EDX made it possible to determine the (qualitative and semi-quantitative) chemical composition on the sample’s surface. The quantities indicated in the EDX microanalysis tables are only semi-quantitative, and serve as an indicator of the quantities of each element present. The microstructure and composition of the porous ceramic were studied under a scanning electron microscope (SEM, HITACHI S-3500N, Ibaraki, Japan). Quantitative analyses were done by an Energy Dispersive X-ray Spectroscopy (EDX) system (Inca-Oxford Instruments, High Wycombe, UK) coupled to the above-described electron microscope using the ZAF (atomic number, absorption and fluorescence) correction software and Bayer standards. Microanalysis data were obtained from the mean of 10 independent determinations. Samples were pre-coated with palladium for the SEM images and were carbon-coated for the EDX analysis in an argon atmosphere using a sputtering machine (Polaron K550X Sputter Coater, Laughton, UK).

#### 4.2.2. X-ray Diffraction (XRD)

This technique consisted of directing an X-ray beam of wavelength 1.5418 Angstroms onto samples to record the presented crystalline phases. With these recordings, the intensities of the diffraction lines that corresponded to the phases were determined and, hence, the present phases could be qualitatively determined. The mineralogical characterization of the powder material was performed by XRD (Bruker-AXS D8Advance, Karksruhe, Germany) by Cu-Kα radiation at 40 kV and 30 mA. Scans were taken with 2 θ values, which varied from 5° to 60° at a rate of 0.05°/min.

#### 4.2.3. Mercury Intrusion Porosimetry (MIP)

Information on sample porosity and pore size distribution was obtained by mercury porosimetry using the Poremaster-60 GT (Quantachrome Instruments, Boyton Beach, FL, USA) within a pressure range between 5.395 KPa and 410,785.062 KPa, which corresponds to a range of pore diameters between 300 μm and 0.0035 μm. 

#### 4.2.4. Statistical Analysis

At least seven runs were performed per sample, and at least three different samples were analyzed per material. The mean and standard deviations were obtained for all the investigated groups. The Kolmogorov–Smirnov test was used to check normality. Comparisons between groups BBM and PBM were made with Student’s t (parametric data) or Mann–Whitney (non-parametric data). All the statistical analyses were performed with the appropriate software (MedCalc v15.8, MedCalc, Ostend, Belgium). Significance was evaluated at a level of *p* < 0.05.

### 4.3. Implant Procedure

#### 4.3.1. Patient Selection and Protocol

Ten partially edentulous patients (five females and five males), with ages ranging from 37 to 60 years and who came to the Department of Oral and Maxillofacial Surgery, were recruited. Patients who demanded fixed restorative appliances in the posterior maxilla were selected for maxillary sinus augmentation due to lack of sufficient bone tissue to place endosseous dental implants. The protocol for harvesting bone samples was approved by the University Ethics Committee (UCAM Ethics Committee; approval ID: 6635) and informed consent was obtained from all the patients. The study was designed following the guidelines set out in the Declaration of Helsinki for experimentation on human subjects. Any possible complications to arise from the surgical therapy were treated following standard dental management protocols.

#### 4.3.2. Inclusion and Exclusion Criteria

The inclusion criteria were as follows: maxillary partial bilateral edentulism involving the premolar-molar areas. The cases with a crestal bone height between 0 and 7 mm and a high postero- lateral atrophy (Cawood V–VI) are most likely to have a two-stage lateral antrostomy. 

The exclusion criteria were: patients suffering an uncontrolled systemic disease or condition known to alter bone metabolism (i.e., osteoporosis, diabetes mellitus, etc.); subjects who were taking/had taken medications known to modify bone metabolism, e.g., bisphosphonates, corticosteroids, etc.; women who were pregnant or trying for pregnancy at the time of screening; patients who presented existing sinus conditions, sepsis, a history of cancer and/or radiation in the oral cavity; complications derived from any of these conditions that affect the sinus area.

#### 4.3.3. Surgical Procedure: First Phase

The study was performed in two surgical phases. In the first phase all the patients took 875/125 mg of amoxycillin/clavulanic acid, every 8 h, starting 1 day before surgery. A dose of 300 mg of Clindamycin every 8 h was prescribed to penicillin-allergic patients. This medication was maintained for 7 days. All the surgical procedures were performed under local anesthesia (Ultracain, Aventis Inc., Frankfurt, Germany). The basic surgical procedure was represented in all the patients by maxillary sinus floor elevation via a lateral approach.

A conventional lateral wall approach was used to perform sinus grafting in all the patients. Fullthickness flaps were elevated to expose the alveolar crest and the lateral wall of the maxillary sinus. A trap door was made in the lateral sinus wall. The sinus membrane was elevated with different shaped curettes until it was completely detached from the lateral and inferior walls of the sinus.

**Figure 9:**
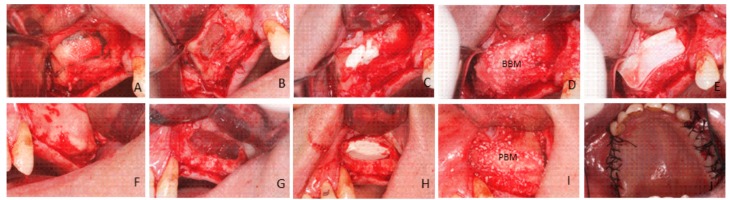
Surgical procedure: (**A**) A crestal incision was made slightly palatally, supplemented by buccal releasing incisions mesially and distally. Fullthickness flaps were elevated to expose the alveolar crest and the lateral wall of the maxillary sinus. (**B**) A trap door was made in the lateral sinus wall. Schneiderian membrane elevation was accomplished by initially exposing and mobilizing the membrane, followed by hand instrumentation to further elevate the membrane along the medial wall of the sinus. The sinus membrane was elevated with different shaped curettes. (**C**) A bioabsorbable collagen barrier membrane was applied underneath the Schneiderian membrane to prevent the dislocation of grafting material if membrane perforation occurred. Randomly, one of the two bone substitute materials was placed into the newly created space between the collagen membrane and the sinus floor (**D**). The bovine porous bone mineral was mixed with venous blood and was packed carefully in the sinus cavity, especially in the posterior and anterior parts (**E**). After bone grafting, a second absorbable collagen membrane was placed over the window (**F**). On the other side, a crestal incision was made slightly palatally, supplemented mesially and distally with two buccal releasing incisions. Full-thickness flaps were elevated to expose the alveolar crest and the lateral wall of the maxillary sinus. (**G**) A trap door was made in the lateral sinus wall using a round bur under sterile saline solution irrigation conditions. The sinus membrane was elevated with different shapes curettes until it was completely detached from the lateral and inferior wall of the sinus. (**H**) A bioabsorbable collagen barrier membrane was placed under the sinus membrane and was adapted to come into contact with the peripheral bony walls. (**I**) The maxillary sinuses were filled with porcine bone material and a short-term absorbable collagen bovine membrane was placed over the window. (**J**) Flaps were sutured.

After membrane elevation, a bioabsorbable collagen barrier membrane was placed under the sinus membrane and adapted to come into contact with the peripheral bony walls (Evolution Fine^®^ OsteoBiol^®^, Tecnoss Dental S.R.L., Torino, Italy). On one side, sinus cavities were grafted with (PBM) (OsteoBiol^®^ mp3, Tecnoss Dental S.R.L., Torino, Italy). After grafting, an absorbable collagen membrane (Evolution Fine^®^ OsteoBiol^®^, Tecnoss Dental S.R.L., Torino, Italy) was placed over the window to minimize soft tissue invasion. On the other side, sinus cavities were grafted with (BBM) (Endobon^®^, RegenerOssTM, BIOMET3i. Palm Beach Gardens, FL, USA). Grafting materials were mixed with venous blood from the defect area and were carefully packed in the created volume following mucous membrane elevation. After bone grafting, a short-term absorbable collagen membrane (Evolution Fine^®^ OsteoBiol^®^, Tecnoss Dental S.R.L., Torino, Italy) was placed over the window. Primary closure was achieved in both cases by suturing with 3–0 silk suture (Laboratory Arago’ n, Barcelona, Spain) (Figure 9).

Antibiotics and analgesics were prescribed for 1 week. All the patients took 875/125 mg of amoxycillin/clavulanic acid every 8 h, starting 1 day before surgery. Moreover, 300 mg of Clindamycin every 8 h was prescribed to penicillin-allergic patients. This medication was maintained for 7 days. Sutures were removed 2 weeks after surgery. During the postoperative period, patients were followed up clinically and radiologically at monthly intervals. 

#### 4.3.4. Surgical Procedure: Second Phase (Bone Biopsy Harvesting)

**Figure 10:**
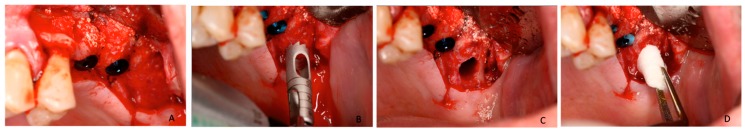
A 3 × 10 mm diameter trephine under sterile saline solution irrigation conditions was used to retrieve a central core of bone upon implant insertion. Sixty bone biopsies were obtained from 60 grafted sites. (**A**) The functional implants were placed on each side; (**B**) 3 × 10 mm diameter trephine under sterile saline solution irrigation was used to retrieve a central core bone; (**C**) each side received three implants; and (**D**) the functional implants were placed in the same sites as the trephined holes on each side.

After a 6-month healing period, the second surgical phase was performed. A 3 × 10 mm diameter trephine, under sterile saline solution irrigation conditions, was used to retrieve a central core of bone upon implant insertion, and 60 bone samples were retrieved for the analysis. Three biopsies were taken from each individual on each slide. Nevertheless, the biopsy value used for the analysis is unique to each individual, and was computed as the mean of the three taken biopsies, thus avoiding possible dependency problems. After retrieval, functional implants were placed at the same sites as the trephined holes on each side (Figure 10).

### 4.4. Sample Processing and Analysis

**Figure 11:**
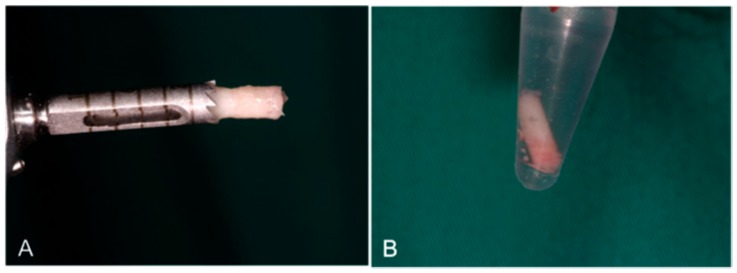
(**A**) A sample of bone core for histomorphometric analysis; (**B**) Sixty bone biopsies were obtained from sixty grafted sites.

Samples were fixed by immersion in 4% formalin solution, dehydrated in a graded ethanol series, and embedded in plastic resin (Technovit A 7210VCL; Kulzer & Co., Hanau, Germany). They were then polished by a manual grinder with 800 grit silicon carbide paper, mounted on an aluminum stub and carbon-coated (Polaron sputter coater). Each block was processed for scanning electron microscopy (SEM). The blocks that contained the entire graft area were obtained using a precision saw. BSE- Back-scattered electron imaging was used to highlight the contrasts among resin, bone and biomaterial. With the image J polygon selection tool irregular shaped areas were outlined by selecting three different regions of interest: bone, residual material and black spaces. All three were calculated in samples of 200 micrometers × 200 micrometers. The images in pseudocolors, obtained, using backscattered electrons, were used to evaluate and measure the histomomorphometric parameters. The selections where outlined in colors, once created this sections where measured by pixels number and was expressed as percentage. A different color was applied to anyone to enhance a difference. The histomorphometric analysis was performed on the bone core samples to determine vital bone content, connective tissue content and residual graft material content (Figure 11).

### 4.5. Statistical Analysis

Quantitative data were recorded as the mean value ± SD. Comparisons between BBM and PBM for each parameter (NB) new bone, (RG) residual graft material and (CT) connective tissue were made with the help of statistical software (MedCalc v15.8, MedCalc, Ostend, Belgium). The conformity of the parameters to normal distribution was assessed by the Kolmogorov–Smirnov test. A Mann–Whitney U test was used for the intergroup comparisons of the parameters without normal distribution. Significance was evaluated at a level of *p* < 0.05.

## 5. Conclusions

The differences found in the physico-chemical characteristics of both xenografts in accordance with the protocol developed on industrial scale justify this distinct in vivo performance. The differences found in porosity, crystallinity and composition terms determine the different behavior of these materials.

The HAs assessed herein are shown to be biocompatible and osteoconductive when used for maxillary sinus elevation purposes. PBM displayed a high level of degradation over the study period. Detailed information about graft material characteristics is crucial to evaluate its clinical outcomes. More histological and histomorphometrical studies are needed to better understand the resorption times of these biomaterials. A more detailed process of resorption of the biomaterials analyzed will be developed in future work.

## Figures and Tables

**Table 1:** Mercury-intruded volume, mode (most frequent diameter) of intraparticle pores, total porosity, and intraparticle porosity of commercial samples.

Materials	Intruded Volume (cc/g)	Mode of Intraparticle Pores (μm)	Total Porosity (%) ^a^	Intraparticle Porosity (%) ^b^
PBM	0.524	0.01–0.004	59.90	38.11
BBM	0.323	0.49	49.13	3.66

^a^ Corresponding to 1 μm < pores < 300 μm; ^b^ Corresponding to pores < 1 μm.
